# Emerging concepts on the anti-inflammatory actions of carbon monoxide-releasing molecules (CO-RMs)

**DOI:** 10.1186/2045-9912-2-28

**Published:** 2012-11-21

**Authors:** Roberto Motterlini, Benjamin Haas, Roberta Foresti

**Affiliations:** 1 INSERM U955, Equipe 3, Faculty of Medicine, University Paris-Est Creteil, Creteil, France

**Keywords:** Inflammation, Carbon monoxide-releasing molecules (CO-RMs), Oxidative stress, Inflammatory mediators, Bactericidal activities

## Abstract

Carbon monoxide-releasing molecules (CO-RMs) are a class of organometallo compounds capable of delivering controlled quantities of CO gas to cells and tissues thus exerting a broad spectrum of pharmacological effects. CO-RMs containing transition metal carbonyls were initially implemented to mimic the function of heme oxygenase-1 (HMOX1), a stress inducible defensive protein that degrades heme to CO and biliverdin leading to anti-oxidant and anti-inflammatory actions. Ten years after their discovery, the research on the chemistry and biological activities of CO-RMs has greatly intensified indicating that their potential use as CO delivering agents for the treatment of several pathological conditions is feasible. Although CO-RMs are a class of compounds that structurally diverge from traditional organic-like pharmaceuticals, their behaviour in the biological environments is progressively being elucidated revealing interesting features of metal-carbonyl chemistry towards cellular targets. Specifically, the presence of carbonyl groups bound to transition metals such as ruthenium, iron or manganese appears to make CO-RMs unique in their ability to transfer CO intracellularly and amplify the mechanisms of signal transduction mediated by CO. In addition to their well-established vasodilatory activities and protective effects against organ ischemic damage, CO-RMs are emerging for their striking anti-inflammatory properties which may be the result of the multiple activities of metal carbonyls in the control of redox signaling, oxidative stress and cellular respiration. Here, we review evidence on the pharmacological effects of CO-RMs in models of acute and chronic inflammation elaborating on some emerging concepts that may help to explain the chemical reactivity and mechanism(s) of action of this distinctive class of compounds in biological systems.

## Introduction

The heme oxygenase enzymes (HMOX1 and HMOX2) generate, among other interesting molecules, the gas carbon monoxide (CO) [1]. The quantity of the gas produced over time depends on the tissue examined, as HO-2 is constitutively expressed in the endothelium, testes and brain while HMOX1 is highly inducible in all tissues by many kinds of stressful stimuli [2]. In addition, the availability of the substrate heme, which is cleaved by heme oxygenases in the α position to release CO, will strictly determine how much CO can be produced in the cell. However, it is expected that CO levels will increase upon up-regulation of HMOX1 and this has been demonstrated in some studies that directly measured CO production ex-vivo [3,4]. Research conducted in the past years has progressively elucidated the role of HMOX1 in mammalian systems. The protein was recognized early on as an important component of the stress response [5,6] but it was only when detailed exploration of the function of its products was performed that an indispensable action for HMOX1 as an anti-oxidant and anti-inflammatory system emerged [7,8]. CO appears to contribute most significantly to these anti-inflammatory activities by regulating a variety of transcription factors, inflammatory proteins and pathways [9-11]. This role is consistent across many inflammatory conditions, although the specific pathways affected might differ from one disease to another.

**Figure 1:**
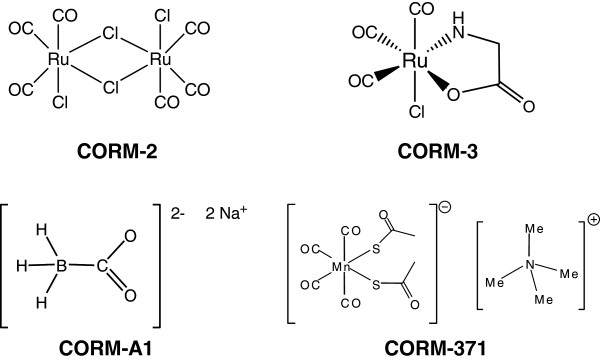
**Chemical structure of the best characterized CO-RMs which have been shown to exert anti-inflammatory and anti-bacterial activities *in vitro *and *in vivo *(see also Table**1**for more details).**

Exposure of cells, tissues and animals to sub-toxic amounts of CO gas has been used successfully to reproduce the anti-inflammatory properties of HMOX1 and has helped to unravel many of the mechanisms underlying this effect [10,11]. The use of CO gas as a therapeutic agent is also underscored by clinical trials currently ongoing in patients who will receive CO by inhalation for the treatment of pulmonary arterial hypertension, post-operative ileus and idiopathic pulmonary fibrosis (see http://www.clinicaltrials.gov). In parallel and as an alternative to this experimental approach, we have focused our strategy on utilizing chemicals that could bind and carry CO stably but deliver the gas when used in biological systems. We have identified and termed these compounds CO-releasing molecules (CO-RMs) [12-14] and have extensively studied their biochemical, biological and pharmacological effects in many *in vitro* and *in vivo* models of disease [9,15-17]. The chemical structure of the best characterized CO-RMs (CORM-2, CORM-3, CORM-A1 and CORM-376) is represented in Figure 1 (see also chemical formula in *List of Abbreviations*). Thanks to strong collaborations with chemists, we have generated CO-RMs with improved water solubility, diverse chemical structures, various rates of CO release and stability [9,15,58-60]. The results have been encouraging as the concentrations and doses of CO-RMs used in all studies were such that the final CO exposure was below the threshold believed to cause toxicity. In addition, as shown in Table 1, the data obtained so far support promising pharmacological actions of CO-RMs that could be useful to counteract inflammatory conditions. Metal carbonyl complexes, containing ruthenium, iron or manganese as metal center, and boranocarbonates are the two major classes of CO-RMs in our portfolio [15,16,59,61]. Our own investigations and studies with collaborators revealed that metal carbonyls are better anti-inflammatory agents than boranocarbonates but the reasons for this difference are unknown at present. The chemistry and pharmacological properties of these compounds is becoming a topic of great interest since in the last 2-3 years other research groups have synthesized a variety of different new CO-RMs. Recently ruthenium imidazole oxime carbonyls [62], photoactive and nanoCO-RMs [63,64], enzyme-triggered CO-RMs [65], CO-RMs encapsulated in micelles [66] and rhenium-based CO-RMs [67] have been developed. However, for most of these new molecules a detailed picture of their behaviour in cells, tissues and *in vivo* models of disease is not yet available, and will be required to evaluate their full pharmacological potential. In this context it is important to emphasize that, once CO is liberated, the potential toxicity of the residual molecule containing the transition metal needs to be carefully evaluated. To date a systematic *in vivo* toxicological profile of CO-RMs has not yet been performed but this will be required once a lead compound will be identified for a given pathological indication.

**Table 1:** **Effect of CO-RMs on *in vitro *and *in vivo *inflammatory disease models**

**Type of CO-RM**	**Inflammatory disease models**	**Effect on inflammatory markers/**	**References**
	***in vitro *and *in vivo***	**Overall outcome**	
CORM-3	Vascular thrombosis in rats	↓ fibrinogen and fibrin	[18]
		↑ pro-thrombin	
CORM-3	Hemorrhagic stroke induced by collagenase in rats	↓ TNF-α production	[19]
		↓ brain injury	
CORM-2	Polymicrobial sepsis induced by cecal ligation and perforation (CLP) in mice	↑ protein C system	[20]
		↓ plasma thrombomodulin	
		↓ number of thrombi in liver, kidney and lung	
CORM-3	Postmenopausal rheumatoid arthritis osteoporosis in mice	↓ cellular infiltration and cartilage degradation	[21]
		↓ TNF-α production	
		↓ Serum levels of IL-6, alkaline phosphatase and	
		MMP-3	
CORM-2	Coagulation and fibrinolitic markers in human umbilical vein endothelial cells (HUVEC)	↓ tissue factor	[22]
		↓ plasminogen activator inhibitor type 1 (PAI-1)	
CORM-2	Neuropatic pain and microglia activation in mice induced by nerve injury	↓ mechanical allodynia	[23]
CORM-3		↓ thermal hyperalgesia	
		↓ nNOS and iNOS expression	
		↓ microglial marker (CD11b/c)	
CORM-3	Vascular inflammation in human umbilical vein endothelial cells (HUVEC)	↓ VCAM-1 and ECAM expression	[24-26]
CORM-2		↓ NF-kβ and p38-MAPK expression	
		↓ mitochondrial respiration	
		↓ NF-kβ and iNOS expression	
CORM-2	Ischemia-reperfusion injury after kidney transplantation in rats	↑ survival rate	[27]
CORM-3		↓ acute tubular necrosis and hemorrhage	
CORM-2	Colitis induced by dextran sodium sulfate in mice	↓ disease activity index	[28]
		↓ myeloperoxidase (MPO) activity	
		↓ TNF-α production	
CORM-2	Endoplasmic reticulum (ER) stress induced by thapsigargin in human hepatocytes and mice	↓ C-reactive protein (CRP)	[29]
CORM-3		↓ serum amyloid P component (SAP)	
CORM-2	Cutaneous wound healing in rats	↑ cell proliferation and wound contraction	[30]
		↑ collagen synthesis	
		↓ TNF-α production and ICAM-1 expression	
		↑ IL-10 production	
CORM-2	Acute hepatic ischemia-reperfusion injury in rats	↑ anti-apoptotic protein Bcl2	[31]
		↓ markers of hepatic damage (AST/ALT)	
		↓ serum levels of TNF-α and IL-6	
		↓ caspase activity and NF-kβ expression	
CORM-2	Acute pancreatitis in rats	↓ serum levels of TNF-α and IL-1β	[32]
		↓ NF-kβ expression and MPO activity	
		↑ IL-10 production	
CORM-3	Rheumatoid arthritis induced by KBxN serum transfer in mice	↑ serum osteocalcin and HO-1 expression	[33]
		↓ MMP-9, MMP-13 and IL-1β expression	
		↓ high mobility group box 1 (HMGB1)	
		↓ Receptor activator of nuclear factor κB ligand (RANKL)	
CORM-2	Ischemia-reperfusion induced inflammation of small intestine in mice	↓ TNF-α and ICAM expression	[34]
		↓ leukocytes rolling and adhesion	
		↓ NF-kβ expression and MPO activity	
CORM-2	Inflammatory response induced by lipopolysaccharide in RAW 264.7 macrophages and BV-2 microglia	↓ iNOS mRNA and protein expression	[35]
		↑ COX-2 expression and NF-kβ expression	
		↑ PGE2 levels	
		↑ phosphorylation of Akt and MAPKs	
CORM-2	Sepsis induced by cecal ligation and puncture (CLP) in mice	↓ neutrophil infiltration in broncoalveolar lavage and liver	[36-39]
		↓ MPO activity in lung and NF-kβ in liver	
		↓ serum TNF-α and high mobility group box 1 (HMGB1)	
		↓ nitrite/nitrate plasma levels	
		↑ cardiac PGC-1α and mitochondrial biogenesis	
CORM-A1	Inflammatory response induced by TNF- α in brain vascular endothelial cells	↓ NADPH oxidase activity	[40]
		↓ apoptosis	
CORM-3	Post-operative ileus in mice	↑ intestinal contractility and transit	[41]
CORM-A1			
		↑ IL-10 and HMOX1 expression	
		↓ IL-6 and iNOS expression	
		↓ leukocyte infiltration	
CORM-2	Inflammatory response in liver and small intestine of thermally injured mice	↓ TNF-α, IL-1β and iNOS expression	[42,43]
		↓ leukocyte infiltration	
		↓ NF-kβ and ICAM-1 expression	
CORM-3	Bacterial activity *in vitro* and bacterial infection *in vivo*	↓ *E. Coli* and *P. Aeuruginosa* growth *in vitro*	[17,44-48]
CORM-2		↑ bacteriostatic and bactericidal effects *in vivo*	
CORM-A1		↑ increased phagocytosis *in vivo*	
CORM-371			
		↓ respiration in bacteria	
ALF-021		↑ survival after bacterial infection *in vivo*	
ALF-062		↓ high mobility group box 1 (HMGB1)	
CORM-3	Collagen-induced arthritis in mice	↓ prostaglandin E2 (PGE2)	[49]
		↓ pro-inflammatory interleukins (IL-1β, IL-2, IL-6)	
		↑ anti-inflammatory interleukins (IL-10)	
		↓ expression of cyclooxygenase-2 (COX-2)	
CORM-3	Vascular inflammation in human neutrophils and rat primary endothelial (EC) and mast (MC) cells	↓ oxidative burst in human PMNs	[50]
		↓ expression of CD54 in rat ECs	
		↓ histamine in MCs	
CORM-2	Inflammatory response induced by pro-inflammatory cytokines in Caco-2 cells and human chondrocytes	↓ prostaglandin E2 (PGE2) and nitrite	[51-53]
		↓ ROS production	
		↓ COX-2 and iNOS protein expression	
		↓ NF-kβ expression	
		↓ metalloproteinase-7 (MMP-7)	
CORM-3	Leukocyte-endothelial interaction *in vitro* and in a models of acute pancreatitis and paw edema in mice	↓ PMNs leukocytes in peritoneal cavity	[54]
CORM-A1			
		↓ PMNs rolling on endothelial cells	
		↓ paw edema and swelling	
CORM-3	Inflammatory response induced by LPS/INFγ and hypoxia in BV2 microglia cells	↓ nitrite and TNF-α production	[55,56]
		↓ hypoxia-reoxygenation damage	
CORM-2	Inflammatory response induced by lipopolysaccharide in RAW 264.7 macrophages	↓ nitrite and TNF-α production	[57]
CORM-3			

In this article we will review the role of CO-RMs in protection against inflammatory conditions, focusing primarily on their effect on oxidative stress and nitric oxide (NO) production, two of the main initiators of the inflammatory cascade. The biochemical and physiological assays used to determine CO liberation or transfer of CO to cellular targets and their interesting anti-bacterial actions will also be discussed.

### Anti-inflammatory actions of CO-RMs

Inflammation and host defense are necessary and intrinsic processes that serve to protect organisms from a series of pathological challenges. The mechanisms that accompany the inflammatory response involve multiple cell types, signalling pathways and transcriptional factors and inflammation appears to be relevant for the vast majority of chronic diseases as well as in acute conditions [68]. That HMOX1 is a key player in mitigating inflammation was first reported in a model of carrageenin-induced pleurisy in rats, in which the evolution of inflammation was accompanied by a dramatic increase in HMOX1 levels and inhibition of heme oxygenase activity enhanced inflammatory markers [69]. In addition, HMOX1 deficiency in human subjects exhibited high levels of vascular inflammation and oxidative stress [70], a finding which is highly reproducible in mice lacking this stress protein [71]. Although bilirubin and biliverdin, endowed with potent antioxidant properties, may be important contributors that fight inflammation [72,73], CO gas applied exogenously is often found to recapitulate many of the anti-inflammatory actions elicited by HMOX1 [74]. Our work on the discovery and characterization of CO-RMs was carried out while novel findings by Otterbein et al. described the powerful effect of CO gas in inhibiting the production of pro-inflammatory cytokines (TNF-α, IL-1β) stimulated by lypopolysaccharide (LPS) *in vitro* and *in vivo*, showing at the same time that CO induced the expression of the anti-inflammatory cytokine IL-10 and that mitogen activated protein kinases (MAPKs) mediated this phenomenon [8]. This and other exciting work stimulated our efforts in the development of CO-RMs and in trying to understand their efficacy in disease models. From an anti-inflammatory perspective, CO-RMs can affect multiple cell types and pathways that coordinate the inflammatory cascade (see Table 1 for a summary of the anti-inflammatory activities of CO-RMs in various *in vitro* and *in vivo* models). For example, Urquhart et al found that CORM-3 strongly reduced neutrophil extravasation in the peritoneum of zymosan-treated mice and inhibited expression of adhesion molecules in human polymorphonuclear neutrophils (PMNs) [54]. Still focusing on PMNs, Sun and co-workers showed that CORM-2 attenuated the leukocyte sequestration, Nfkβ activation and endothelial protein expression of ICAM-1 in the lung of thermally injured mice [75]. The multiple effects of CO-RMs were particularly well dissected in a study by Masini et al. where human PMNs primed to elicit an inflammatory response were co-incubated with rat endothelial cells or perivascular mast cells [50]. Here the authors clearly showed that CORM-3 down-regulated the oxidative burst in PMNs, the over-expression of adhesion molecules in PMNs and endothelial cells and the release of histamine and up-regulation of an activation marker by mast cells. These results indicate how CORM-3 modulates acute inflammation by reducing the activation of PMNs, the first responders in host defense, but also by inhibiting expression of molecules and inflammatory factors that perpetuate the inflammatory process. In RAW macrophages and BV-2 microglia we have also shown concentration-dependent decreases in nitrite and TNF-α production by CORM-2 and CORM-3 following challenge with LPS [55-57].

**Figure 2:**
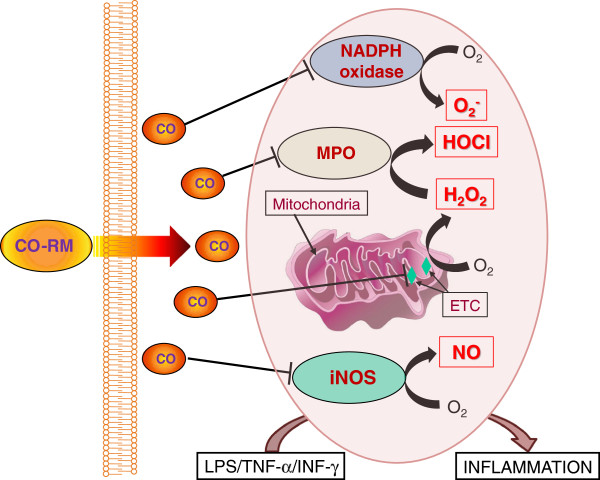
Graphical representation summarizing the mechanism(s) potentially involved in the anti-inflammatory activities of CO-RMs (see text for details).

**Figure 3:**
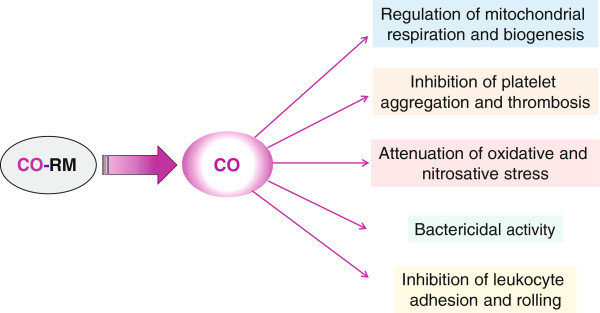
**Schematic diagram representing the diverse effects of CO liberated from CO-RMs *in vitro *and *in vivo *(see text for details).**

The *in vivo* anti-inflammatory action of CO-RMs has also been consistently described. The group of Alcaraz has performed a series of detailed investigations in arthritis models [21,33,49] and demonstrated that daily treatment with CORM-2 or CORM-3 can effectively suppress the clinical and histopathological manifestations of disease. Levels of PGE-2 and many other inflammatory mediators were reduced in the joint and this resulted in a better preservation of cartilage tissue and bone structures [33]. However, modulation of inflammatory molecules levels surely is not the only mechanism contributing to the CO-RMs mediated protection against inflammation and the data by Lancel and colleagues point to mitochondria as very important cellular organelles that are affected by CO-RMs. In a model of sepsis induced by cecal ligation, CORM-3 administration conserved cardiac mitochondrial function by preventing sepsis-mediated damage to mitochondria thus preserving membrane potential and respiration and inducing mitochondrial biogenesis [36]. In the heart of mice fed a high fat diet to mimic a metabolic syndrome-like disorder CORM-3 also stimulated mitochondrial biogenesis [76]. The mode of action and efficacy of CO-RMs may also depend on the timing of administration in relation to the pathology studied, as exemplified recently by our investigation in a model of hemorrhagic stroke in rats [19]. Indeed, we observed that CORM-3 pre-treatment (5 min) or post-treatment (3 days) of rats after the onset of the hemorrhage elicited protective effects while administering the compound 3 hours after the stroke, in correspondence to the acute phase of the disease process, resulted in exacerbation of damage. The striking observation of this study is that one single dose of CORM-3 could modify the long-term inflammatory scenario that followed the hemorrhagic stroke by redirecting and limiting the infiltration of peripheral leukocytes and neutrophils in the brain and reducing the local activation of brain microglia and astrocytes induced by the stroke. Importantly, CORM-3 appeared to finely tune the levels of TNF-α, by allowing its positive action in reparatory processes but inhibiting its detrimental effects. Thus, a growing body of literature supports a beneficial role of CO-RMs in inflammatory models but future investigations are required to better establish their therapeutic applications (see Figures 2 and 3 for the proposed mechanism of action of CO-RMs *in vitro* and *in vivo*).

### Mechanisms underlying the effects of CO-RMs on inflammation: modulation by oxidative stress

It is well accepted that inflammatory stimuli promote a variety of responses that participate to exacerbate damage in cells and tissues but also promote the resolution of inflammation. Oxidative stress, derived from excessive and persistent production of reactive oxygen species (ROS) and a possible decrease in antioxidant defences, accompanies or precedes the increased amounts of inflammatory mediators upon inflammatory challenge. Because CO has a high affinity for different heme-containing proteins – cytochromes in mitochondria and NADPH oxidase in the cell [77] - that contribute to regulate the levels of ROS, it is intriguing that part of the anti-inflammatory activities of CO-RMs may derive directly from inhibiting the generation of these damaging (or signalling) species. CO-RMs have shown a tendency to modulate pathways that produce ROS and the chemical nature of transition metal carbonyls might favour this reaction by allowing a selective transfer of CO from the CO-RMs to the target [78,79]. In RAW macrophages treated with LPS or PMA-stimulated neutrophils CORM-2 inhibited NADPH activity and overproduction of superoxide anion (O_2_^−^) [80]. Similarly, CORM-A1 reduced the accumulation of ROS induced by TNF-α in pig cerebral microvascular endothelial cells, possibly by acting on a specific subunit of NADPH oxidase (Nox4) which is highly expressed in these cells [40,81]. Notably, a reduction in oxidative stress was reported also in chondrocytes from cartilage specimens of patients suffering from osteoarthritis, emphasizing both the relevance of these findings in primary human tissue and the idea that pathological processes occurring in diseased tissue can be modified by application of CO-RMs [51]. Oxidative stress levels were also significantly reduced by CORM-3 in intestinal tissue in a clinically relevant model of postoperative ileus and this was accompanied by partial restoration of antioxidant capacity levels [41]. Increased production of ROS following TNF-α/cycloheximide exposure was also diminished by CORM-A1 in a mouse intestinal epithelial cell line [82]. In summary, different CO-RMs can inhibit ROS/oxidative stress that results from inflammation, thus affecting an early and crucial mechanism that modulates subsequent inflammatory processes (see Figure 2).

### Mechanisms underlying the effects of CO-RMs on inflammation: modulation of inducible nitric oxide synthase (iNOS) and NO production

Inflammation is a complex phenomenon; thus, it is anticipated that any anti-inflammatory properties of CO-RMs would involve a number of metabolic pathway. Overproduction of NO following up-regulation of inducible NO synthase (iNOS) is a critical step in the initiation and propagation of the inflammatory response [83] and diverse actions of CO-RMs in relation to this system have been described although with quite conflicting findings. We have observed that CORM-2 and CORM-3 decrease NO levels produced by macrophages stimulated with LPS without affecting iNOS protein expression [57] and because of these results we have postulated that CO from CO-RMs inhibited the activity of iNOS, a heme-containing protein already shown in purified form to be blocked by CO gas [84]. Similar results were obtained in microglia by Min KJ et al. [85] while Megias and colleagues actually demonstrated that iNOS expression was reduced by CORM-2 in Caco-2 cells challenged with a combination of IL-1*β*, TNF*-*α and IFN*-*γ [52]. Other authors have reported the same observation in the gut [41] and in the spinal cord [23] following inflammatory damaging conditions, strengthening the idea that indeed CO-RMs exert an inhibitory effect on iNOS induction and activity. This would not perhaps be surprising if we consider that CO-RMs seem to affect the activation of Nfkβ [24,25,52], which controls the expression iNOS and is a master regulator of major pathways in inflammation. However, until detailed studies designed to dissect the effect of CO-RMs on activity versus induction of iNOS are performed, it will not be clear whether CO-RMs can inhibit NO generation or iNOS expression. It may well be that inhibition of both can occur simultaneously or that one effect or the other will prevail depending on the inflammatory condition, tissue analyzed and the type of CO-RM investigated.

### Bactericidal activities of CO-RMs

The threat of bacterial infection is omnipresent in surgical settings, wounds and contaminated food, any of which may lead to fatal consequences. Interestingly, CO-RMs have been shown to possess anti-bacterial properties that may be among the important therapeutic applications envisioned for this class of compounds. Lack of HMOX1 in mice resulted in an exaggerated lethality after cecal ligation and puncture (CLP), which caused polymicrobial sepsis [86]. However, the administration of CORM-2 was able to increase phagocytosis, decrease circulating bacterial counts, and rescue HMOX1^–/–^ mice from the exaggerated mortality of CLP-induced sepsis, even when applied 6 hours after the initiation of infection. This is a remarkable result that emphasizes how these molecules can exert pleiotropic actions in such a complicated and severe pathological scenario. Desmard et al. also demonstrated that CORM-3, CORM-2 and, to a lesser extent, CORM-371, exert anti-bacterial actions against *P. Aeruginosa in vitro* and *in vivo*[17,87]. The ruthenium-based CO-RMs appeared more effective and CORM-A1 only exerted a transient bacteriostatic action, highlighting again the importance of the metal in mediating some activities of CO-RMs and perhaps guiding CO to the appropriate cellular target. More detailed work has been performed to investigate the direct effect of CO-RMs on different bacteria and the results have been reviewed elsewhere [88,89].

Using more biochemical-oriented approaches, it has been possible to determine that terminal oxidases are targeted by CORM-3 when inhibiting bacterial growth [17,44], thus impairing bacterial respiration. Moreover, oxidative stress caused by CO-RMs is another factor explaining some mechanistic actions of these compounds. According to Tavares et al., exposure of *E. Coli* to CORM-2 or a molybdenum-containing CO-RM increased the levels of intracellular ROS as well as causing DNA damage and disruption of Fe-S clusters [45]. The authors also showed that thiol-based antioxidants prevented the anti-microbial properties of CO-RMs, a finding we corroborated in studies using *P. Aeruginosa*[17,87]. However, in our work no effect of CORM-2 or CORM-3 on ROS production was detected as assessed by the use of a fluorescent probe [17], while in another collaborative investigation it was observed that thiols reduced the ROS production stimulated by CORM-2 in *P. Aeruginosa* biofilms but that this reduction was not accompanied by inhibition of bacterial growth [46]. Thus, the collective evidence suggests that CO-RMs interacts with metal-based proteins present in bacteria to exert various effects that are related to their bactericidal or bacteriostatic properties. However, it is possible that other pathways, susceptible to changes following application of CO-RMs, contribute to CO-RMs-mediated anti-microbial effects. In this regard, data obtained from microarray analysis of bacterial genes affected by exposing *E. Coli* to CORM-2 and CORM-3 have provided fascinating information about the pathways responding to CO-RMs [44,90]. It should be noted that one study looked at aerobically and anaerobically grown *E Coli* and CORM-2 while the other investigated CORM-3 in anaerobically grown *E Coli*, thus already indicating that different results should be expected from this analysis. Saraiva and colleagues have nicely summarized the diverse, and perhaps still incomplete, information collected in these two transcriptomic approaches showing that some pathways are typically changed in anaerobic conditions, some only in the aerobic state and some are instead commonly altered in *E Coli* grown either in aerobic or anaerobic conditions [88]. Of interest, genes involved in zinc homeostasis and the bacterial response to oxidative stress (SoxRS and OxyR) were increased in both conditions, perhaps stressing that, irrespective of the growth medium, the presence of ruthenium in CO-RMs and their propensity to cause oxidative stress/inhibit the respiratory complexes will consistently occur. The fact that genes modulating biofilm pathways are affected is also a clear signal that *E. Coli* is suffering from exposure to CO-RMs and thus attempts to boost its resistance to these agents by promoting biofilm formation. In addition, increased methionine metabolism is triggered by CO-RMs, which may still be linked to an oxidative stress response.

In summary, few, but well designed and informative reports support the idea that CO-RMs are useful compounds to be employed alone or in combination with other antibiotics [46] to fight bacterial infection, taking into account the important notion that the bactericidal actions of CO-RM-s are elicited at concentrations that do not harm mammalian cells [17].

### Liberation of CO by CO-RMs: biochemical and pharmacological assays

Assessing CO liberation from CO-RMs has been a priority since our discovery of these compounds. Initially we developed a myoglobin assay for the detection of carbon monoxy myoglobin (MbCO) and employed an amperometric CO electrode to determine the rate and quantity of CO released [12,16]. Gas chromatography techiques have also been used by others to assess the spontaneous liberation of CO from CO-RMs in solution. In parallel, we used bioassays such as relaxation of aortic vessels and inhibition of the inflammatory response in macrophages to assess the CO-mediated pharmacological effects of CO-RMs [12,57,91]. In most cases we found a very good correlation between the rate and mode of CO release by CO-RMs and their effect on aortic ring relaxation. The results from several studies have also enabled us to propose that the chemical structure of metal carbonyls CO-RMs might facilitate the direct transfer of CO from CO-RMs to the intracellular target(s) as it appears that the release of CO from certain metal carbonyls (i.e. CORM-2 and CORM-3) requires an acceptor [17,44,92]. This may enhance selectivity for the action of CO from metal carbonyls and the difference with CO gas applied exogenously would be that its diffusion into cells might be limited or hampered by the encounter of many proteins potentially able to bind CO, including the prototypic intracellular target(s) [17,77]. Although this concept needs to be substantiated, results on the bactericidal effects of CO-RMs (see above) and an interesting article published recently reports data in this direction. Wang and colleagues have developed a genetically encoded fluorescent probe, which is capable of selectively detecting CO inside living cell [93]. The probe, named COSer for CO sensor, consists of a permuted yellow fluorescent protein inserted into the regulatory domain of the bacterial protein CooA, a heme-dependent transcription factor known to bind CO with high affinity and selectivity. It was found that the fluorescent intensity of HeLa cells transfected with COSer increased after addition of 5 μM CO gas and a higher response was obtained with 10 μM. Interestingly, the fluorescence intensity was even stronger in cells treated with CORM-2 as a very significant response was obtained with only 1 μM CORM-2 and to obtain a given fluorescence intensity, more CO gas was needed with COSer-transfected cells than with the purified probe. These findings led the authors to state that CORM-2 provided an alternative and more controllable method for CO delivery to cells and could have possibly reduced the difficulty they encountered in getting CO into cells by using simple CO solutions. Similar findings were recently obtained by Michel and co-workers, who have synthesized a palladium-based fluorescent probe that is capable of detecting CO with high selectivity both in aqueous solutions and in living cells. Notably, CORM-3 was used in their experiments as the source of CO revealing that, unlike CO gas, concentrations as low as 1 μM CORM-3 were sufficient to trigger fluorescence in cells loaded with the palladium probe [94].

The use of the MbCO assay to assess the rate and amount of CO liberated by CO-RMs has been recently questioned [95]. In our experiments we showed that while MbCO is promptly formed after addition of CORM-2 or CORM-3 to a solution containing reduced Mb, a sensitive CO electrode failed to detect any CO upon addition of these two CO-RMs [17]. However, CO release from CORM-A1, a boranocarbonate, is detected by the Mb assay and by the electrode with comparable results, indicating the spontaneous liberation of CO from the compound. McLean and colleagues have shown that in the case of CORM-3 and CORM-2 liberation of CO and the consequent formation of MbCO is facilitated by dithionite, which is usually added in excess to the assay for keeping Mb in a reduced state [95]. The authors concluded that the MbCO assay should be abandoned and propose the use of hemoglobin (Hb) since it binds CO with a much greater affinity than oxygen and does not require deoxygenation by dithionite. We believe these data indicate that dithionite and other sulphite can accelerate the release of CO from CO-RMs and that rates of CO release obtained with the MbCO assay should be interpreted cautiously, but we would like to add few important considerations still in favour of the MbCO assay. First, the results by McLean and colleagues seem to imply that liberation (or transfer) of CO from CORM-2 and CORM-3 to a prototypic target (i.e. Mb) cannot occur in the presence of a deoxygenated reduced heme but is triggered only by interaction with anions such as sulphites. That this is not the case is elegantly described by Obirai and colleagues in an interesting report published few years ago revealing quite the opposite as CORM-2 was demonstrated to directly transfer CO to a heme(FeII)/heme(FeIII) redox couple [92]. Using a cyclic voltammetry method, the authors proved that when CORM-2 is added to an argon deaerated phosphate buffer solution containing an electrode coated with the heme redox couple but in complete absence of dithionite, a heme(FeII)-CO complex is formed. Secondly, the determination of the rate of CO liberation from CO-RMs by using an *in vitro* biochemical assay is rather approximate and we always judged it best to interpret our data on CO liberation using a combination of approaches as these compounds are designed for their possible therapeutic use *in vivo*. That is the reason why in our studies on the characterization of CO-RMs we always coupled the quantification of CO liberation *in vitro* with data obtained using bioassays that reflect more closely the behaviour of these compounds in complex biological systems. For instance, despite the fact that CORM-2 and CORM-3 are stable compounds in solution and may not liberate CO spontaneously, they still cause a rapid relaxation in isolated vessels and hypotension in animals suggesting that these compounds are fast CO releasers *in vivo* in line with the MbCO assay data. The bioactive effects mediated by the rapid CO release from these two CO-RMs have been corroborated by using pharmacological tools (i.e. inactive CO-RMs or CO-RMs depleted of CO) [15,57,91] or by comparison with compounds that release CO much slower *in vitro* and *in vivo* (i.e. CORM-A1 and CORM-371) [16,17]. As a further example, we have recently employed the MbCO assay to determine that CORM-401, a manganese-containing CO-RM, liberates 3-4 CO per molecule [60]. We found that the relaxation exerted by this molecule in aortic rings is approximately 3-fold more pronounced than that elicited by the same concentration of CORM-A1, which has a half-life similar to CORM-401 but liberates only 1 CO (unpublished results). Third, the use of oxygenated Hb instead of reduced Mb poses other relevant issues, such as the presence of 4 hemes and the co-operative effect of CO binding to the hemes, which will make it more difficult to quantify the amount and kinetics of CO released. These considerations, together with the results of the MbCO assay and the recent findings with the fluorescent probes reported above, strongly indicate that the release of CO from CORM-2 and CORM-3 occurs when the metal carbonyl is in the vicinity of a reduced iron acceptor (MbFe(II) or heme(II)). The results have also important implications on the efficacy of metal carbonyl CO-RMs in delivering CO to prototypic intracellular targets.

## Conclusions

Although the initial discovery of CO-RMs took place a decade ago [12], it is intriguing that many diverse and novel pharmacological actions are being discovered for these compounds. The studies conducted so far reflect a real effort to understand the biochemical mechanisms that mediate the beneficial effects of CO-RMs. The emerging scenario is that, while CO-RMs mainly affect cellular functions via the liberation of CO, the molecules may facilitate or modulate other concomitant reactions involving redox and metal-sensitive pathways. Importantly, the chemical reactivity of metal carbonyl complexes-based CO-RMs may be enriching, rather than diminishing, their positive actions suggesting that a critical assessment of CO-RMs’ behaviour in biological environment (bioassays) must always be evaluated in parallel to their ability to release CO. In view of the growing importance of inflammatory components in initiating and modulating pathological processes, we have focused here on how CO-RMs modulate the inflammatory response as consistent and converging data point to their interesting anti-inflammatory activity. As new CO-RMs with a multiplicity of chemical properties and reactivity are being synthesized and tested in biological models, we may in the future uncover novel promising applications for this unique class of compounds.

## Abbreviations

CLP: Cecal ligation and puncture; CO: Carbon monoxide; CO-RMs: Carbon monoxide-releasing molecules; CO: Carbon monoxide; CORM-2: [Ru(CO)_3_Cl2]_2_) also known as tricarbonyldichlororuthenium(II) dimer; CORM-3: Ru(CO)3Cl(glycinate) also known as tricarbonylchloro(glycinato)ruthenium(II); CORM-371: [Me_4_N][Mn(CO)_4_(thioacetate)_2_]; CORM-A1: NaH_3_BCOOH, also known as sodium boranocarbonates; COX-2: Cyclooxygenase-2; Hb: Hemoglobin; (HMOX1): Heme oxygenase-1; ICAM-1: Intercellular adhesion molecule 1; IL: Interleukin; IL-1β: Interleukin 1 beta; iNOS: Inducible nitric oxide synthase or NOS(III); LPS: Lypopolysaccharide; MAPKs: Mitogen activated protein kinases; Mb: Myoglobin; MbCO: Carbon monoxy myoglobin; MMP: Matrix metallo proteinase; MPO: Myeloperoxidase; NO: Nitric oxide; Nfkβ: Nuclear factor kappa beta; O_2_^−^: Superoxide anion; PGE2: Prostaglandin E2; PMNs: Polymorphonuclear neutrophils; ROS: Reactive oxygen species; TNF-α: Tumor necrosis factor alpha.

## Competing interests

Dr. Roberto Motterlini was founder and scientific director of hemoCORM (2004-2008), holds patents on the CO-RMs technology and is a shareholder of Alfama.

## Authors’ contribution

RM and RF wrote the manuscript. BH drafted an earlier version of the manuscript and helped with the outline of Table 1. All authors have read and approved the manuscript.
